# Activistic citizenship in nursing homes: co-ownership in the mundane

**DOI:** 10.1177/14713012231155307

**Published:** 2023-01-30

**Authors:** Marianne Sund, Halvor Hanisch, Kirsten Jaeger Fjetland

**Affiliations:** Centre of Diaconia and Professional Practice/Faculty of Health studies, 87446 VID Specialized University, Stavanger, Norway; Work Research Institute, 60499 Oslo Metropolitan University, Oslo, Norway; Faculty of Health Studies, 87446 VID Specialized University, Stavanger, Norway

**Keywords:** nursing home, dementia, co-ownership, activistic citizenship, silence, narrative resistance, narrative analysis, ethnography

## Abstract

The traditional narrative of dementia, focused on cognition as constructive of personhood, has been challenged by person-centred care as well as a rights-based citizenship lens. However, reports of everyday discrimination leading to occupational deprivation and pathologising interpretations of people living with dementia in nursing homes highlight the need for further investigation. The purpose of this study was to investigate the transformative power of mundane and relational enactments of citizenship in nursing homes, exploring the potential of adding an activistic lens of citizenship to our interpretive practices. Through an ethnographic study in Norwegian nursing homes, a narrative analysis of fieldnotes and interview transcripts was conducted. Narratives were interpreted using narrative theory, occupational perspectives and theories of citizenship. Findings reveal a phenomenon of shared ownership between residents and staff, and a vulnerable balance between silence and active social and occupational engagement in the nursing homes. Further, they shed light on how group-based assessments of residents’ abilities or occupational needs may constrain opportunities, and staffs’ options, to facilitate co-ownership. We suggest that a lens of activistic citizenship implies interpreting residents’ behaviours as mundane forms of subtle resistance. A professional and ethical responsibility building on such interpretive practices may turn attention towards structures that constrain residents’ expressions of citizenship.

## Introduction

Some stories achieve a master status in society, embodying the culture’s shared understandings. Their power comes from their invisibility as they become taken for granted (McKenzie-Mohr & Lafrance, 2017). The traditional narrative of dementia focused on cognition as constructive of personhood, silencing people’s stories based on the belief that it was impossible to access their experiences (Baldwin, 2008). Such reductionist views have been challenged, for example through the lens of person-centred care (Kitwood, 1997) and, more recently, the promotion of citizenship (Bartlett et al., 2010; Hydén & Antelius, 2017; Nedlund et al., 2019). Person-centred care acknowledges the unique perspectives and needs of people with dementia (Kitwood, 1997), while the rights-based lens of citizenship sheds light on socio-political factors that impact people’s opportunities for practising citizenship (Bartlett et al., 2010).

In Norway, people with dementia are entitled to quality, professional services (Norwegian Ministry of Health and Care Services, 2001), in private homes or nursing homes with 24-hour staffing. The current Norwegian dementia plan (Norwegian Ministry of Health and Care Services, 2020) promotes the engagement of people with dementia within a dementia-friendly society, whilst reporting that many living in long-term care experience loneliness and lack meaningful everyday experiences. Building on research documenting its significance for wellbeing, the plan explicitly promotes person-centred care as the grounding principle of dementia services and guidelines. Citizenship and strategies to ensure residents’ influence on the organisation of nursing homes are scarcely addressed.

While the Convention on the Rights of Persons with Disabilities (CRPD; United Nations, 2006) promotes people’s entitlements to the same liberties as others in society, dementia rarely features in disability reports or research (Cahill, 2018). According to Ward et al. (2016), while headline stories of neglect or abuse may be featured in public discourse, minor narratives of repetitive and mundane everyday discrimination may pass unseen. For example, residents in nursing homes may be at risk of occupational deprivation (Du Toit et al., 2019; Morgan-Brown et al., 2019), experience feelings of captivity and homesickness (Heggestad et al., 2013), find life boring (Mjørud et al., 2017) or adapt to its routines (Eyers et al., 2012). People with dementia living in this context are also at risk of being interpreted through pathology (Dupuis et al., 2012; Steele et al., 2020), in turn preventing them from challenging power relations within long-term care settings.

Citizenship scholars in the field of dementia have promoted understanding citizenship as practices rather than a status that is bestowed. Nedlund et al. (2019) argue that to understand everyday citizenship we must analyse how relationships (between citizens, the state and its institutions) can change and are interrelated in practice. A conception of everyday citizenship recognises agency as key to securing social equality, while encapsulating its dimension on rights (legal connection to society), access (conditions for practising citizenship) and belonging (legal and subjective). Baldwin and Greason (2016) suggest that citizenship can be realised through engagement in mundane everyday activities, while Morgan-Brown et al. (2019) argue that citizenship entails an obligation to identify and address occupational injustice. However, Sund et al. (2022a) conclude that further research is needed on how citizenship can be realised in a way that encompasses both residents’ needs and abilities as citizens in the nursing home. In a recent article, Sund et al. (2022b) argue that citizenship in this context is not a stable condition, but emerges in fragile moments of *becoming*, in both embodied and relational ways. Such moments shed light on residents’ abilities for growth and for acting in line with their own occupational identity. Building on such conceptions, this article aims to explore the transformative power of such mundane and relational enactments of citizenship, asking: *How can mundane social and occupational situations in nursing homes shed light on citizenship for people with dementia, and what is the potential of adding an activistic lens of citizenship to our interpretive practices?*

## Theoretical Framework

This section outlines the article’s main theoretical lenses. The first lens perceives humans as occupational beings, highlighting our inherent need for *doing*, in line with our own sense of self (*being*), experiencing *belonging* to people and places, and continued opportunities to grow and develop (*becoming*) (Wilcock & Hocking, 2015).

The second lens is the concept of narrativity, through theories acknowledging humans as narrative beings. Our understanding of narrative theory acknowledges the actions, movements and expressions of residents in nursing homes as narrative agency (Baldwin, 2008), enabling the recognition of diverse ways in which residents express their Selves and desires, and enact influence towards their environment. In line with Fjetland and Gjermestad (2018), citizenship is perceived as a relational, expressive and narrative phenomena, while narrative competence is crucial to understand and *interpret* active aspects of citizenship. Such interpretive practices imply expecting people’s expressions, whether verbal or non-verbal, to be intentional.

Our third lens is concerned with citizenship, in which Boje (2017) distinguishes between ordinary, active and activistic citizenship. Ordinary citizenship means people practise their citizenship through their daily routines, while active citizenship is characterised by people participating in the public sphere, within the frame of the democratic system’s structures and rules of engagement. Activistic citizenship contributes to formulating and re-forming the economic, social and political conditions that set the frames for social life. The activistic citizen fights for equality, solidarity and justice, and can be characterised by citizens enacting civil disobedience in the form of breaking with pre-conditioned rules and regulations. However, Neveu (2015) argues that citizenship can be found in practices that challenge norms, habits and established patterns in mundane aspects of life. Through ‘acts of citizenship’ (Isin, 2008) that rupture the given or habitual, people can become claimants of citizenship in unexpected ways.

While these three lenses have been combined in this study, they have also been slightly modified. Following Ursin and Lotherington (2018), we underline that doing depends upon different material and social actors as well as knowledge regimes. Our lens of citizenship is extended to encompass people in vulnerable life situations. This extension moves slightly beyond Boje’s (2017) argument that citizens must act and actively claim citizenship in the public realms of society, and instead recognises the narrative (Fjetland & Gjermestad, 2018) and everyday aspects (Nedlund et al., 2019) of citizenship.

## Methods

This article is part of a PhD project exploring what characterises citizenship practices in nursing homes, through an iterative–inductive ethnographic research design (O’Reilly, 2009). Inspired by interpretive (hermeneutic) phenomenology (Wright-St. Clair, 2015), we sought to explore mundane, often taken-for-granted aspects of everyday living in nursing homes.

### Recruitment and Study Context

Two nursing homes in the south-west region of Norway participated in the study. Municipalities were recruited through written invitations to leaders in several municipalities, to which two responded positively. Leaders within the municipalities chose which nursing homes to include. The main inclusion criteria were that units should be intended for long-term stay for people with dementia. All residents with a dementia diagnosis living in the included units were eligible, and invited, to participate. Sea-Crest and Sunny Hill were located in different municipalities, comprising one large unit and two small units, respectively. Leaders and staff at both nursing homes expressed an explicit aspiration of providing person-centred services to residents.

#### Sea-Crest Nursing Home

Fieldwork took place at Sea-Crest nursing home during the spring of 2019. The nursing home was located in a rural area in a large municipality. One large unit (twice the size of those at Sunny Hill) on the second floor was included. Some residents did not have a diagnosis of dementia, resulting in 10 residents being part of the study. The unit was divided into two groups, each with its own living room, access to a small terrace, a small kitchen, and a dining room area. The doors to the unit were not locked.

#### Sunny Hill Nursing Home

Fieldwork took place at unit North and unit South at Sunny Hill from November 2019 to January 2020. The nursing home was located in a medium-sized municipality. The two units were the same size; however, North was reserved for people in need of close supervision in everyday life, locked via code to prevent residents from leaving on their own (with extra staffing). South had an open door. Some new residents moved in during fieldwork, resulting in 16 residents participating in total. Both units were situated on the ground, with direct access to outdoor areas, and were equipped with a small kitchen, dining room tables, and a living room area where residents gathered.

### Data Collection

To explore mundane aspects of nursing home life, the first author (hereby referred to as the researcher) conducted participant observation, in-situation conversations, individual interviews and group interviews (see Table 1 for an overview of the data). These methods enabled observation of the activities and routines of the units, as well as talking to residents and staff about their experiences and perspectives of everyday life.

**Table 1.** Overview of gathered data.

	Fieldwork duration	Number of visits	Field-notes	Residents involved	Interviews with staff	Group interviews
Sea-crest	36 h.	16	62 p.	10 (9 women and 1 man)	5	1 (2 participants)
Sunny hill north	42 h.	14	57 p.	9 (all women)	5	1 (4 participants)
Sunny hill south	45 h.	15	60 p.	7 (all women)	3	1 (3 participants)

#### Participant Observation

Seeking to understand phenomena from participants’ perspectives required time to build trust between the researcher, residents and staff (Antelius et al., 2018). The participant observation took place within the nursing homes’ common areas, whereas the researcher participated in activities and routines—during the day, in the evenings and on weekends. She spent time talking to and observing staff; however, the majority of her time was spent sitting beside residents, engaging in conversation or observing everyday life as it unfolded. At the end of each day of fieldwork, the researcher wrote detailed, chronological fieldnotes documenting happenings, activities, expressions and researcher reflections. A total of 179 pages of fieldnotes constituted the main material of analysis for the article.

#### Interviews and Group Interviews

During fieldwork, the researcher found that residents had difficulty understanding the research taking place. Given that cognitive challenges can make abstract thinking or eliciting past experiences more difficult (Nygård, 2006), she chose not to conduct audio-recorded sit-down interviews with residents, instead focusing on in-situation conversation that enabled residents to express their views in naturally occurring situations. Audio-recorded interviews with staff members took place after a period of observation. Informed by fieldnotes, they were asked about situations the researcher had observed, organisational structures and routines, and their perceptions and experiences of the nursing home. A semi-structured interview style (O’Reilly, 2009)—continuously focused throughout the fieldwork and adjusted according to participants’ roles—allowed flexibility in each conversation. Fieldwork culminated with an audio-recorded group discussion with staff from each unit, discussing preliminary interpretations and experiences that emerged throughout the study.

### Ethical Considerations

The Norwegian Regional Ethics Committee determined that the study was not subject to the Norwegian Health Research Act (Norwegian Ministry of Health and Care Services, 2009), and the study was given dispensation from professional secrecy because of its observational nature. Approval was granted by the Norwegian Centre for Research Data, and the study was conducted in accordance with the National Committee for Research Ethics in the Social Sciences and the Humanities’ (NESH; 2021) guidelines for research ethics. Information about the project was provided orally and in writing. Residents were informed by nursing home staff or next-of-kin, and tailored written information was provided by the researcher. In line with the NSD approval and the (2021) NESH guidelines, signed consent forms were obtained from participants (the residents, staff and leaders). Staff at Sea-Crest signed consent forms before fieldwork started; at Sunny Hill they signed during their first meeting with the researcher. As residents had difficulty understanding the concept of the research taking place, next-of-kin were asked to sign the consent form on the residents’ behalf; in some instances, both the resident and next-of-kin signed. All consent from residents/next-of-kin was obtained by nursing home staff before the researcher met the residents. Data were stored on encrypted hard drives, separate from participants’ information. All participants were given pseudonyms.

### Narrative Analysis

Although we originally aimed for a conventional ethnographic analysis, initial readings demonstrated that we needed increased methodological sensitivity to the unique expressive acts of the residents. Instead of producing knowledge of the whole social structure of the nursing homes (Robson, 2002), we turned attention towards the particular characteristics of residents’ stories. While narrative analysis is typically used in relation to verbal performance and interview data (Squire et al., 2013), we built upon an understanding of humans as narrative (Baldwin, 2008) and occupational (Wilcock & Hocking, 2015) beings, thus viewing both verbal storytelling and performative practices as narrative acts (Hydén & Antelius, 2011). In this way, the narrative approach was able to encompass observational data from ethnographic fieldwork. Polkinghorne (1995) distinguishes between analysis of narratives, where stories are used to produce common categories and themes across the dataset, and narrative analysis, where researchers uses a wide array of data to produce coherent stories. We chose to do the latter, giving prominence to residents’ verbal and enacted expressions as ways of communicating stories of meaning and identity. Since these constructed or synthesized narratives should not be confused with spontaneous narratives in primary data, we call them emplotted narratives.

**Figure 1.**
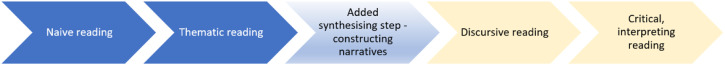
Steps of analysis.

The analysis was inspired by the four analytical readings suggested by Fjetland (2015):

1) naïve reading, 2) thematic reading, 3) discursive reading and 4) interpretive reading. In addition, a synthesising step of constructing narratives (Polkinghorne, 1995) was added. The analytical steps are now briefly outlined (see Figure 1):

*Step 1*: *Naïve reading*. Fieldnotes were read several times, asking ‘What are fieldnotes about, and how is citizenship characterised in the stories?’ This focused our gaze towards the ways residents acted and interacted within the social environment of the nursing homes.

*Step 2*: *Thematic reading.* Fieldnotes were re-read, seeking to identify recurring themes and activities happening in the units. The thematic reading did not produce specific and common categories, but brought forward themes throughout the various datasets, leading to the added step of narrative construction.

*Step 3*: C*onstructing narratives.* This synthesising step—constructing emplotted narratives, meant generating small and coherent stories from diachronic data, drawing events and actions together into unified episodes (Polkinghorne, 1995). This enabled unique and unfolding narratives of how residents acted within their social environments to become the focus of our interpretations.

*Step 4*: *Discursive reading*. After the narrative construction, we interpreted challenges and opportunities for citizenship within the stories and within their unique contexts. Interview transcripts were searched to identify staff members’ perceptions and expressions that could shed light on the interpretations.

*Step 5*: *Critical, interpretive reading.* The narratives were now interpreted in terms of the article’s theoretical framework, asking: What is the potential of adding an activistic lens of citizenship to our interpretive practices?

## Findings and Interpretations

Everyday life in the nursing homes seemed to follow a predictable temporal structure. The staff were generally active, interacting with residents or engaged in daily practical tasks. Sea-Crest provided a varied program of activities for residents, planned by a designated activities organiser. At Sunny Hill, residents participated in some activities outside the units, but most happened within their social environments, initiated by staff and residents. In the units, residents spent much time gathered in the common areas, sometimes reading newspapers, watching television, engaging in conversation, singing, listening to music, exercising or joining organised activities. However, many residents spent a great deal of time sitting silently without interacting with others, sitting with their eyes closed or fell asleep. Residents’ need for support varied. Some required physical help, e.g., support while walking or assistance with feeding. Some verbally and in embodied ways expressed uncertainty, anxiety, discomfort or sadness, and were observed seeking physical contact, such as a hug, sitting close or holding hands. Some residents waited and made no demands, while others clearly stated their satisfaction with staff and the services they received. At times, someone was also heard verbally expressing frustration because they could not leave or did not understand what was happening, or because of too much waiting or not enough to do.

Through analytically engaging with narratives of ordinary social life derived from fieldwork, a phenomenon of shared ownership emerged between residents and staff, born from situations where residents acted and interacted in agentic ways, while at other times they *seemingly* dis-engaged. Such phenomena are now explored through a set of narratives constructed from fieldnotes taken at Sea-Crest and Sunny Hill nursing homes.

### Co-Ownership Through Being and Doing Together

One of the narratives that gave rise to the analytical phenomenon of co-ownership was a social situation involving Monica, May and Jasmine, three residents living at unit South at Sunny Hill nursing home:

It is Sunday, shortly after breakfast. Monica is sitting in her regular recliner. There is a radio on a small table next to her, playing Christmas songs. Monica says she loves listening to Christmas music. She controls the radio, regulating the volume several times, depending on what is playing. May is seated on the couch; she exclaims ‘Listen to the nice music!’ several times, and moves her arms in rhythm with it, as if she’s conducting. Jasmine has just finished eating and is walking towards the living room with a staff member. The staff member says to Jasmine that she can choose where to sit. May waves at Jasmine, and says she can come sit with her. Jasmine sits down on the couch next to May, and they have a little chat. May exclaims, ‘Listen to the nice music!’, and again conducts with her arms. Jasmine looks at her, smiles and says, ‘Or like this’, and makes movements as if she’s playing electric guitar. They both laugh.

Observing residents’ interactions gave the researcher an impression of togetherness between them, through finding common ground in which to interact. The radio Monica was controlling had large buttons, intuitive for her as it was likely similar to the type of radio that was normal in her younger years. When the staff member told Jasmine to sit wherever she wanted, this opened up a social situation where May invited Jasmine to sit with her, just like any friends joining each other for a social gathering. The *s*taff member was barely present in the narrative—simply as someone walking alongside, having no active part in the events that unfolded. Still, she was close by, something that staff member Marit stressed the importance of during her interview: ‘it’s important that staff is in the living room to, in a way, observe that they are okay and don’t say bad things to each other. Of course, that can also happen—someone can gang up on another and be rude’. The researcher did observe situations where residents’ interactions led to frustration. For example, one day May was frowning, asking if something was wrong, because she repeatedly misunderstood what another resident was saying. When staff joined them and translated what was said, May became relaxed again. Staff at South expressed an explicit goal of presence within the social environment and therefore rarely exited the living room area at the same time. This active choice of presence seemed to enable them to support residents’ attempts at social engagement as they naturally occurred.

Taking a closer look at this social situation sheds light on how the micro community of the nursing home could support residents to actively influence each other in a meaningful way, as citizens. Monica took charge of the musical entertainment, providing joy and a topic of conversation for the others. May contributed to an inclusive environment by inviting Jasmine into the situation, creating an opportunity for social interaction. While this narrative of social ownership may at first glance appear to be an independent endeavour, our interpretations suggest otherwise. If we view citizenship as a collective achievement (Ursin & Lotherington, 2018), both the intuitive design of the radio (as a material actor) and the presence and support of the staff (as social actors) contributed to residents’ opportunity to engage in such a way. In addition, one might view nearing Christmas as an actor in itself, which enabled engagement by providing a theme that residents held in common. An occupational perspective (Wilcock & Hocking, 2015) recognises that such *doing* alongside others fosters *belonging*, through a sense of being in the right place, recognised by others, having a place in the social world. Experiencing a sense of belonging, as a citizen, can be seen as a central dimension of citizenship (Nedlund et al, 2019). In this narrative, such belonging appeared enabled by a shared ownership between residents and staff.

### Co-Ownership and the Phenomenon of Silence

At Sea-Crest, staff appeared to have limited time to spend in such ‘informal’ social situations, due to a range of medical responsibilities and tasks requiring their attention. When residents were not engaged in organised activities, mealtimes or one-on-one interactions with staff, they were often observed sitting beside each other, as they did this Monday, seemingly without interacting:

Tove, Kåre and Lisa are sitting in the living room. The TV is showing a Danish programme in which cars are being auctioned off. Kåre is seated in a recliner and Tove is sitting on the sofa next to him; no one speaks. Kåre takes out his wallet several times, looks at the cards, puts the cards back in his wallet, and places the wallet in his pocket. Lisa is seated by the door, some distance from the others. She is not watching the television. A new programme starts; it’s ‘Poirot’, in English with Norwegian captions. No one pays attention. Lisa closes her eyes, slumps over to the side. Klara enters in her wheelchair, propelling it with her arms and feet. She stops next to the television, watching it briefly before falling asleep. She slumps over to the right, in a position that looks uncomfortable. No one speaks.

Residents sat beside each other in the living room for 45 min, without speaking to each other, while staff were occupied in other tasks. Staff told the researcher that residents had difficulty engaging with each other without support; however, their other responsibilities often meant they had limited time to provide it. Staff member Anna stressed the importance of staffs’ presence: ‘We can see that, if we’re not sitting in the living room, then the atmosphere isn’t always very good. Therefore, they need someone to be there, who can vary what is said and lead some conversations’. Being present this day, and during other similar situations, left the researcher with a sense of *silence*—not because of a lack of sound, but due to the absence of interaction. In Fivush’s (2010) article, she distinguishes between *being silenced*, as something imposed, and *being silent*. While imposed silence signifies a loss of power, *being silent* can be a shared experience, a silent attunement of being together or a restful and quiet time of reflection. Occupational theory (Wilcock & Hocking, 2015) similarly recognises the human need for occupational balance, between our *doings* and our sense of *being*, through time for stillness, rest and reflection. Sitting beside others in silence might represent such shared moments.

*Being silenced*, on the other hand, happens through actual silencing, not allowing the speaker to talk, or by one’s voice not being given credibility (Fivush, 2010). In a study by Holthe et al. (2007), residents were found to be reliant on staff to engage in their social environment. Not having such support might be perceived as a form of *silencing*. While being occupational does not equal being continuously active, both occupational (Wilcock & Hocking, 2015) and citizenship (Bartlett et al, 2010; Nedlund et al, 2019) theory highlight people’s right to live in environments where they have the opportunity for engagement. Meanwhile, residents living in nursing homes might be at risk of social and occupational deprivation—as exemplified by Morgan-Brown et al. (2019), who report that in their study 62% of residents’ time in the communal rooms was spent dis-engaged. The master narrative of dementia might further normalise dis-engagement as apathy, perhaps as something that does not require as much attention: ‘Some behavioural symptoms, like apathy, may create few challenges for the environment, while other symptoms, such as aggression […] can create larger challenges for the environment. It is important that services are arranged to handle behavioural incidents’ (Norwegian Ministry of Health and Care Services, 2020, p. 69, my translation).

Our use of the term *silence* depicts situations where residents stopped making observable communicative sound and ceased interacting with each other. Seemingly, when residents were left full ownership and autonomy of the room, their actual ownership decreased. Acknowledging the narrativity of other expressions then the spoken word (Baldwin, 2008; Hydén & Antelius, 2011), we recognise that if this ‘voice’ is expressed both verbally and through the things we do, then *being silenced* relates to both the opportunity to speak and the opportunity to act. While silence might be moments of rest and attunement, or imposed through lack of support or recognition, might it also be interpreted as an expression of citizenship? According to Isin (2008), acts of citizenship rupture the given. They indicate a doing but do not necessarily involve the motion of objects or bodies. As such, they might be easily overlooked. Throughout the fieldwork, the researcher saw that Kåre, Tove, Lisa and Klara were all quite capable of expressing themselves verbally and engaging in conversation; however, having the ability does not necessarily equate to having the opportunity. The ‘act’ of withdrawal—of silence, such as sitting for 45 min beside each other without interaction—might actually be interpreted as an expression of agency, a shared silence of resistance towards environments that do not sufficiently support social engagement. While attempting to interpret the meaning of silence may be fraught with uncertainties, such a perception stands in contrast to diagnostic perceptions, accepting *silence* as symptoms (apathy), and thus a natural consequence of dementia.

### Decreasing Co-Ownership Through Routines

Meals were arguably the most recurring activity within all three nursing home units. They seemed to create a predictable frame of reference for both residents and staff, and each unit had their own routines for how meals were conducted. At Sea-Crest, residents were served their meals in a regular fashion:

It’s Sunday at nine. Several residents are seated at the dining table, waiting to be served. There are no food or drinks on the tables. When staff is ready, they serve preprepared sandwiches and each resident chooses which one they want. Several choose jam sandwiches, and everyone is offered boiled eggs. The plate of sandwiches is then placed on a trolley positioned near the kitchen counter. One staff member gives out medication. Another helps Nelly eat. A third staff member is seated on a stool over by the trolley. None of the residents speak to each other during the meal.

Residents were served sandwiches, poured drinks and given medication during breakfast as part of their regular routine. During her individual interview, staff member Pia talked about the routines: ‘But, I feel like it’s so strict, in a way, with these routines all the time. That it should—everyone in for breakfast at nine, and if it’s not like that it becomes a bother, almost’. She wanted more flexibility, so residents could help set the table, serve, make their sandwiches; in other words, she wanted them to be able to do more of the things they were used to doing. During her individual interview, staff member Nina explained that most wouldn’t be able; ‘“…most can’t. So that’s where it comes from, starting to make it for them, actually. But maybe someone might want to make them. I haven’t thought to ask’. In her interview, Renate similarly expressed: ‘But I can’t imagine that they, that those we have in now would be able to make them. They have, in a sense, enough with just eating those sandwiches’.

Serving breakfast, ensuring that no food was placed within arm’s reach of residents, left the room as the staff’s domain. This routine of serving appeared to be founded on a group-based assessment concluding that residents’ limited abilities would make them unable to contribute more actively. While research suggests that rooms and occupations associated with food are important for feelings of *home* in nursing homes (Dekker & Pols., 2020; Heggestad et al., 2013), the kitchens and areas associated with food often seemed to be the domains of staff during fieldwork. For example, no residents in any of the units were observed helping themselves to dinner; all were served by staff from the trolley or kitchen counter. In an article contemplating multiple forms of silence in narrative inquiry, Blix et al. (2021) write that Sami children, in response to Norwegian assimilation policies, learned to story themselves as Norwegian, by silencing parts of who they were. They argue that this silencing was also a silencing of resistance. Might one similarly, on a micro level of nursing home life, interpret residents as storying themselves to ‘fit’ with what is expected of them? This is similar to Boyle’s (2008) argument that residents internalised assumptions of dependency rather than openly resisting them, and Eyers et al.’s (2012) study, where residents changed previous everyday habits to adapt to the care home setting. Adapting to the routines and expectations of the nursing homes may lead to a silencing of part of oneself. Although residents did not verbally express discontentment, viewing mealtimes through an activistic lens underlines that group-based assessments of residents’ abilities may constrain their opportunities, and staffs’ options, to facilitate co-ownership. Hammell (2020) writes that though many people have the ability to engage, they also need the opportunity. While residents being left full ownership of the room might decrease their actual ownership, staff taking full ownership through assessing residents as unable to contribute seemed to have similar results. There might be benefits from building on interpretive practices that assess residents as capable of agency and support that agency by sharing responsibility.

### Resisting Calmness and the Act of Taking Ownership

Residents were sometimes observed acting in ways that might be interpreted as attempting to claim occupational ownership in the nursing home. The following brief narratives features Hilde and Alma, who both had moved to unit North quite recently:

It’s Friday afternoon. Several residents and staff are gathered in the living room. Hilde gets up from her chair and leaves for the corridor. One of the staff follows, and they return together soon after. The staff member says, ‘We can’t go out today. It’s so cold outside’. Hilde seems restless. She looks around the room, moving her upper body and arms slightly, while sitting. Shortly after this, she walks towards the kitchen with a glass, saying it’s going in the dishwasher. A bit later a staff member brings a pile of clothes to the table in the living room, which several residents help fold—most remaining seated. Hilde is standing by the table, thoroughly stretching the clothes before folding them. The clothes are quickly folded, and the staff member gathers the clothes to put them away. Hilde brings a pile and follows staff to the linen cupboard on her own initiative. She returns to the living room and takes out her knitting, but soon starts moving restlessly in her chair. A short while later, Hilde gets up and walks towards the kitchen. She knocks and the staff member opens the door. Hilde hands a spoon to her, saying that it needs to go in the dishwasher.

Another day, Alma is walking around the unit, adjusting tablecloths and chairs that are askew. She walks over to me, straightens my scarf and picks a stray hair from it, before gently stroking my hair. She then moves towards the dining area again, where staff are working. One of the staff asks her if she wants to sit down in the living room and relax. She follows her into the living room and Alma sits down with her. A moment later, Alma gets up and approaches another staff member, who is cleaning the tables. The staff member gives Alma the cloth, and she washes both tables.

During fieldwork, staff members at unit North explained that they had an explicit aspiration for calmness and ‘sitting activities’ in the living room, because too much movement or activity could lead residents to become uneasy or frustrated. However, they regularly invited residents to participate in more active activities, such as the balloon game or folding clothes. At the same time they made sure that the activities did not last long, to prevent residents from becoming overstimulated. As staff member Charlotte said during her interview, ‘There aren’t that many in a way, that many different activities that we can do with them. They do need calm and they are tired, so they also need to rest. So, it’s not about doing something all the time’. Staff explained that one staff member should always be seated with residents. If not, residents might begin to move about, becoming agitated. The researcher did observe this effect—for example, a resident could start searching for home if staff were not there to provide a sense of safety, give reassurance or explain what was going on. However, sometimes, such as in the narrative of Alma, the strategy seemed to be applied by default, encouraging her to ‘sit down’ even though her current actions seemed to be pleasurable for her.

Staff told the researcher that Hilde was used to being active, going for long walks and working actively in the home. Alma was also used to the responsibility of household chores, and staff explained that she did not like messes. Their actions may be seen as a way of performing their story (Hydén & Antelius, 2011) and occupational identity (Wilcock & Hocking, 2015). Although staff recognised their occupational identities, the routine of preventing overstimulation at times seemed to overshadow this knowledge. In their article, Capstick and Chatwin (2016) demonstrate how residents engaged in a form of cultural resistance through a range of strategies, as a way of ‘answering back’ (p. 172). Institutional aspects of nursing homes can pathologize such strategies or resistance as challenging behaviour (Steele et al., 2020), and as such neglecting the intention or power that might be assigned to it. Residents taking the initiative to engage in occupations or arenas over which staff normally held ownership might instead be viewed as attempts at claiming ownership through mundane acts of citizenship (Isin, 2008). If the essence of an act is that it is a rupture in the given (Isin, 2008), then Hilde attempting to leave the living room or approach the kitchen, or Alma acting against staff’s encouragement of rest, might be seen as a break with staff’s group-based aspirations for calmness. Viewing such actions as a mundane form of subtle resistance sheds light on residents’ ability to perform their citizenship, claiming ownership within the nursing home.

## Discussion

Capstick and Chatwin (2016) argue that biomedical and psychosocial discourse largely focus on the passive role of the person with dementia ‘as the “spoken to”, “spoken for”, “spoken about”; he or she is constructed as dependent, needy, and lacking in agency’ (p. 172). This expresses concern with the notion of personhood as *bestowed*, also implying that it can be revoked. In contrast, an activistic lens of citizenship, as suggested in this article, recognises that residents’ expressions can hold transformative power. Through co-ownership, we do not merely *bestow* residents with meaning or opportunity, we recognise that their *acts* continuously demonstrate how we can support their efforts to practise their citizenship. This requires what Fjetland and Gjermestad (2018) term a professional competence in understanding and actively interpreting residents as citizens.

Activistic citizens make a difference by questioning established roles (Neveu, 2015). This connects the activistic lens to change. Interpreting the actions and expressions of residents at Sea-Crest and Sunny Hill, we do not suggest that residents aimed to be activistic. We suggest that in this context, where residents rely on the support and interpretive practices of the nursing home and its staff, they are also at the mercy of them. If we are to bridge the gap between residents’ needs and capabilities as citizens, we need to recognise multiple knowledge perspectives (Sund et al., 2022a), including medical, care-based and citizenship theories. Sund et al. (2022b) suggest that one way to do so is to recognise that residents’ growth as occupational beings and citizens in nursing homes can emerge in embodied and relational ways, shedding light on residents’ need to live in environments that support their natural and embodied desires to *do*. Our understanding of activistic citizenship links such conceptions with a more transformative ambition of change of both contextual as well as relational perspectives in professional practice, which connects directly with our interpretive practices.

While active and activistic aspects of citizenship and political influence are normally centred on public arenas of society (Boje, 2017), residents in our field study did not engage in these realms, nor were they expected to. However, as argued by Volpp (2017), when the activities of home become objects of government, they then become sites for contesting citizenship. The nursing *home*, as a politically willed context, is deeply ingrained in the political. While Donaldson and Kymlicka (2017) argue that the underlying moral purpose of citizenship means being responsive towards all citizens’ desire to control and shape their world, people with dementia risk being viewed as not able to know, or express, their own views and needs (Steele et al., 2020)—and are thus prevented from asserting influence. However, for people living in nursing homes, attempts at influence may unfold in indirect and unspoken ways (Mondaca et al., 2018), and take habituated, embodied and emotional forms (Boyle, 2014), thus requiring particular sensitivity and attention to be identified. According to Fjetland and Gjermestad (2018), exploring active citizenship in the context of profound intellectual disability, interpreting agency entails expecting expressions to be intentional. This sheds light on how recognising residents’ opportunities for influence, and thus change, relies on staffs’ interpretive practices.

Through recognising, following the CRPD (United Nations, 2006), that residents have the right to live a life as others do and to participate fully, silence or acts against group-based assessments can be interpreted as resistance towards routines that constrain such citizenship practices. Co-ownership as a phenomenon underlines the possibilities of a shared ownership between residents and staff in nursing homes. Paying attention to the expressions of residents as possible ‘acts of resistance’ may contribute to pointing the way forwards. However, for such mundane acts of citizenship to have transformative power requires a professional and ethical responsibility by staff to interpret residents as intentional citizens and to promote change.

## Conclusion

The findings of this article highlight a phenomenon of shared ownership between residents and staff, and a vulnerable balance between *silence* and active social and occupational engagement in the nursing home. Through a lens of activistic citizenship, group-based assessments of residents’ abilities or occupational needs are interpreted as constraining opportunities, and staffs’ options, to facilitate co-ownership. Interpreting silence or attempts at taking ownership as acts of citizenship turns attention to the risk of pathologising mundane forms of subtle resistance. We suggest that an activistic lens of citizenship recognises residents’ expressions as holding transformative power. This transformative lens appears directly linked to how interpreting residents as intentional and capable shows us ways to support their efforts to practise their citizenship.

### Strengths and Limitations

The current article presents results from an ethnographic study in three nursing home units in Norway, interpreting the particular characteristics of selected narratives observed during fieldwork. While the sample under study was limited to two nursing homes in a specific Norwegian region, the results may have relevance for other similar contexts—not for their direct transferability, but by suggesting ways one can utilise a citizenship lens to question the logics and structures of nursing home services. However, the reader should be aware that interpreting meaning from ethnographic participant observations may be fraught with uncertainty, and findings must therefore be treated with a certain degree of caution.

Our design limited our focus to public and semi-public areas of the nursing home; as we opted to respect the residents’ integrity, this limited our opportunity to include the more personal aspects of residents’ citizenship. Since almost all residents in the nursing homes where fieldwork took place were female, differing perspectives and citizenship enactments from a male point of view may be missing.
